# *Erratum:* Vol. 65, No. 15

**DOI:** 10.15585/mmwr.mm6736a7

**Published:** 2018-09-14

In the report “Notes from the Field: Respiratory Symptoms and Skin Irritation Among Hospital Workers Using a New Disinfection Product — Pennsylvania, 2015,” in the fourth paragraph, the fourth sentence should have read “Full-shift air sample results for hydrogen peroxide ranged from **<11** parts per billion (ppb) to 511 ppb; for acetic acid, from **<8.8 ppb to 319.4** **ppb**; and for peroxyacetic acid, from **<2.2** ppb to 48 ppb.”

